# Effect of Salt Addition and Fermentation Time on Phenolics, Microbial Dynamics, Volatile Organic Compounds, and Sensory Properties of the PDO Table Olives of Gaeta (Italy)

**DOI:** 10.3390/molecules27228100

**Published:** 2022-11-21

**Authors:** Raffaele Sacchi, Giandomenico Corrado, Boris Basile, Daniele Mandarello, Maria Luisa Ambrosino, Antonello Paduano, Maria Savarese, Nicola Caporaso, Maria Aponte, Alessandro Genovese

**Affiliations:** Department of Agricultural Sciences, University of Naples Federico II, Via Università 100, 80055 Portici, Napoli, Italy

**Keywords:** *Olea europaea*, table olive, SPME-GC/MS, polyphenols, microorganisms, VOC, fermentation, PDO

## Abstract

‘Oliva di Gaeta’ is almost certainly the most important and well-known PDO denomination for table olives in Italy. Their production is based on a specific two-stage trade preparation called the ‘Itrana’ method. In this work, we investigated how variations in the duration of the initial water fermentation (i.e., 15 and 30 days) and the salt concentration (i.e., 6% and 8% NaCl) influence the chemical features, microbial dynamics, polyphenols, volatile organic compounds, and sensory features of ‘Oliva di Gaeta’. The time of the addition of salt did not affect the final concentration in the brine, but a longer initial water fermentation (before salt addition) led to lower pH values. The bacterial count constantly increased until the salt addition (i.e., either 15 or 30 days), while the yeast population peaked on day 30. Generally, the two different salt concentrations did not affect the count of microorganisms at the end of fermentation, with the only exception being a higher lactic acid bacteria count for the treatment with 6% salt added at 30 days. At commercial maturity, the crucial bitter tastant oleuropein was not completely removed from the drupes, and differences in salt concentration and the length of the first-stage water fermentation did not influence its content at the end of olive curing. Richer volatile profiles of olives were detected with higher-salt treatments, while the combination of low salt and early saline treatment provided a more distinct profile. Longer initial water fermentation caused a small increase in some phenolic compounds (e.g., iso-verbascoside, verbascoside, and hydroxytyrosol-glucoside). A panel test indicated that salt application at 30 days resulted in a more “Sour” and “Bitter” taste, irrespective of the salt concentration. The low salt concentration coupled with the late saline treatment resulted in more “Fruity” notes, probably due to the higher production of esters by lactobacilli. The slightly bitter perception of the olives was consistent with the partial removal of oleuropein. Our work revealed the characteristics of the ‘Itrana’ method and that the variation in salt concentration and its time of application changes parameters ranging from the microbial dynamics to the sensory profile. Specifically, our data indicate that 6% NaCl coupled with a longer initial water fermentation is the most different condition: it is less effective in blocking microbial growth but, at the same time, is more potent in altering the nutritional (e.g., polyphenols) and sensorial qualities (e.g., bitterness and fruitiness) of ‘Oliva di Gaeta’.

## 1. Introduction

Olives are an archetypical food of the Mediterranean diet, and their widespread use in gastronomy dates to the dawn of Western civilization. Globally, most of the olive production is used to extract oil, while around a tenth is employed to obtain table olives [1]. Raw olives are unpalatable, mainly because of the high content (14% dw) of oleuropein [2,3], a bitter-tasting glycosylated seco-iridoid involved in the defense against the olive fruit fly (*Bactrocera olea* Rossi) [4]. Different procedures have been developed for debittering, and many are based on soaking in water, saline solutions, alkalis, or their combinations [5,6]. The storage of raw olives in salt water is considered the most probable original system because of the large availability of sea water in the traditional area of the cultivation of the olive tree. The use of a dilute alkaline solution for debittering started to become more diffuse in the XIX century with the development of the chemical industry as an independent sector, and it represents one of the first uses of lye to cure food [1,7]. Prolonged storage in water-based solutions is associated with considerable microbiological fermentation, which mainly involves lactic acid bacteria (LAB) (e.g., *Lactobacillus plantarum* and *L. pentosus*) and yeasts (e.g., *Saccharomyces cerevisiae*, *Wickerhamomyces anomalus*, and *Candida boidinii*) [5]. Fermentation not only favors debittering but also enriches the gustatory profile [8]. Microbial fermentation, for example, produces a wide range of volatile compounds, which provide a specific aroma to table olives [9,10,11].

In a simplistic way, the alteration of olive acidity is probably the most relevant chemical process during fermentation, and it is mainly caused by LAB [12,13]. The biochemical processes occurring during olive fermentation are, however, much more complex and are affected by a wide range of variables, such as duration, the type and concentration of microorganisms, salt concentration, and temperature, as well as the maturity stage of the fruit and its phenolic content [1,5,14,15].

Table olives are largely produced and consumed around the Mediterranean basin, with Italy being the EU country with the third highest production. A characteristic of Italian production is that olives are cured using traditional methods that have been tailored to the plethora of local varieties that typify the Italian olive sector [16,17]. Currently, different Italian table olives are listed in the so-called “EU quality register” (https://ec.europa.eu/info/food-farming-fisheries/food-safety-and-quality/certification/quality-labels/geographical-indications-register accessed on the 1 October 2022), and they are covered by EU Protected Designation of Origin (PDO) denominations for geographical indication. These table olives are highly appreciated by consumers and represent a valuable element for safeguarding the import–export balance of the olive trade [18].

‘Oliva di Gaeta’ is arguably one of the most important and well-known PDO denominations for table olives in Italy, as also indicated by the fact that this trade name is loosely used to indicate medium–large-sized olives of deep-pink-to-purple color, with an easily detached pulp, a soft texture, and a desirable slight bitter taste. The PDO denomination can be applied only to olives of the ‘Itrana’ variety, a dual-purpose late-ripening olive almost exclusively cultivated in the Lazio and Campania regions of Italy. This variety is said to have originated in the village of Itri (Latina, Italy). Fermentation is achieved with the so-called ‘Itrana’ method, a traditional process whose origin is anecdotical. Briefly, olives are picked when ripe (starting from the third week of March) and quickly transferred to tanks filled with water to favor rapid natural fermentation. Subsequently, marine salt is added to stabilize the process, with continuous monitoring to replenish water (and salt if necessary). This curing method, as in many other processes used in the table olive sector, is still craft-based. They all have the intrinsic risk of variable-quality production and of an unstable final product. These uncertainties are typically managed empirically [19].

The need to match consumer demand for standardized and safe traditional table olive products requires a deeper understanding of the effects of the technical factors employed to drive fermentation. This is also necessary to increase the sustainability of the process, for instance, by reducing the use of salt and other resources without affecting olive palatability [19]. Moreover, concerns about the health risks associated with excessive sodium consumption [20] drove several countries to introduce salt reduction strategies [21], which include the establishment of lower sodium content targets for common foods. Although the contribution of table olives to daily sodium intake is limited, using lower-than-usual or lower-than-optimal salt concentrations during the manufacturing of table olives may be also appreciated by consumers from a dietary perspective.

Relatively little effort has been made to study these issues in relation to specific traditional fermentation procedures if compared with the more diffuse Spanish, Greek, or Californian trade preparation methods. Previously, the chemical–physical, nutritional, and sensory analysis of ‘Itana’ olives indicated that the traditional method produces very different olives from those obtained with Greek-style fermentation [22]. Moreover, while the role of chemical, biochemical, and microbiological factors in table olive maturation has been widely addressed, studies that integrated these investigations with a panel-based sensory analysis are more limited for traditional preparation methods, because many studies have often been focused on the role of starter cultures [5,23].

For these reasons, the aim of this work was to assess, for the first time, the impact of the main processing conditions during the spontaneous fermentation of ‘Itrana’ olives to (i) correlate the changes with the physical and chemical characteristics of the final product and (ii) propose appropriate conditions that can guarantee a suitable nutritional and sensorial profile. Specifically, we studied the effects of the duration of the initial water fermentation time (i.e., 15 and 30 days) and those of the salt concentration (i.e., 6% and 8%) by analyzing the main chemical parameters, microbiological dynamics, phenolic profile, and sensory characteristics of the final product.

## 2. Results and Discussion

### 2.1. Evolution of pH and Salt Concentration of the Brine

At harvest, the olives had a mean length and diameter of 21.5 ± 1.6 mm (mean ± s.d.) and 17.4 ± 1.0 mm, respectively. The mean fruit fresh weight was 4.1 ± 0.8 g (79.5 ± 3.0% of which was fruit flesh), whereas the moisture content was 51.8 ± 0.7%. Soon after harvest, the olives were used for preparations in plastic containers.

**Figure 1:**
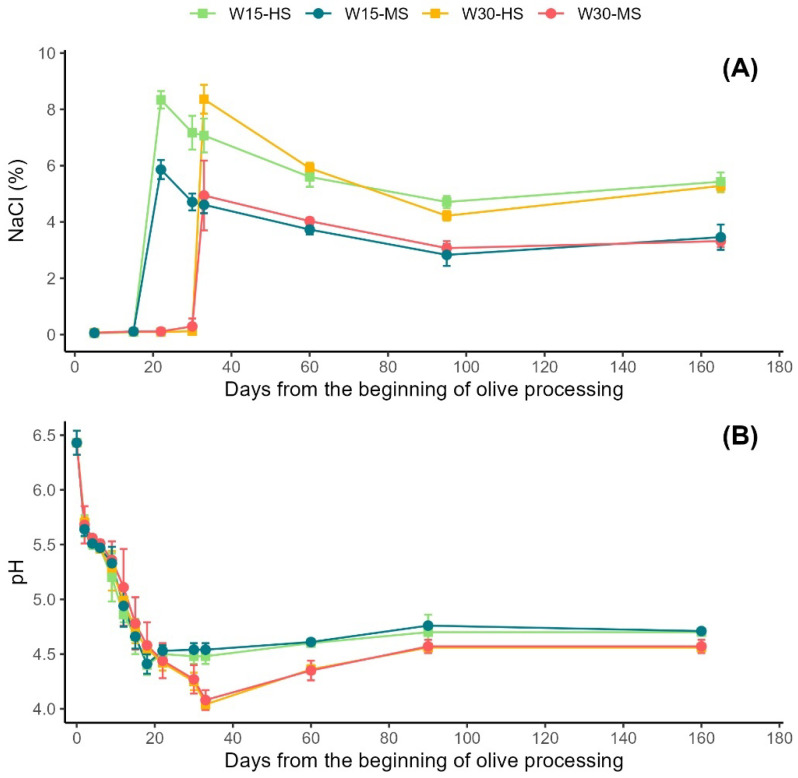
Evolution of (**A**) sodium chloride concentration and (**B**) pH of the brine during four processing methods. W15-HS: 15 days in water and then salting with 8% NaCl brine (pea); W15-MS: 15 days in water and then salting with 6% NaCl brine (teal); W30-HS: 30 days in water and then salting with 8% NaCl brine (orange); W30-MS: 30 days in water and then salting with 6% NaCl brine (crimson).

The effects of the treatments on the evolution of the NaCl concentration and pH of the brine during olive fermentation are reported in Figure 1.

As expected, the salt concentration was lower during the whole process when using the 6% NaCl dose instead of the standard 8% NaCl. For each dose, the time of the addition of salt (either on day 15 or day 30 after the start of olive processing) did not affect the final salt concentration, whose values were stable in the second half of the process. Brine acidity, as indicated by the decreasing pH value, quickly increased in the first two weeks, and it was stabilized by the addition of salt. The pH difference between the minimum values for W15 and for W30 was limited. Differences between the W30 and W15 treatments after the salt addition became less pronounced with time. The pH did not change significantly in any samples after approximately day 40 of fermentation. After that, the brine of W30-HS and W30-MS treatments had higher pH values than the W30-HS and W30-MS samples. Irrespective of the saline concentration, the pH decreased to 4.5 on days 18–22 without further significant variation for samples starting saline fermentation on day 15. On the contrary, in the samples that underwent the longest first-stage fermentation (W30-HS and W30-MS), the pH dropped to about 4.1. Following day 90, the pH of the brine became stable, and significant differences were not observed between any treatments at the end of the storage.

Overall, a longer initial water fermentation (before salt addition) led to lower pH values. Nonetheless, at the end of the curing process, the main acid products reached equilibrium among treatments. Considering the evolution of the brine pH and of the differences between 6% and 8% salt concentrations, the data suggest that variations in the final product are likely caused by the early stage of olive processing.

The effect of the salt addition on olive fermentation, as well as on other food products, probably evolved to prevent spoilage by harmful microorganisms, in addition to improving the taste and texture of the drupes. The reduction in the use of sodium salts during olive debittering has gained attention because of dietary concerns and the need to increase sustainability [19]. In terms of the pH of the brine, both saline doses were equally effective in controlling acidification, without differences at the end of the fermentation process, even for the late addition of the lower dose. It should be added that the employed difference in salt concentration is not large in absolute terms (minus 2%). Studies on table olives of the ‘Itrana’ variety and particularly on the effects of the factors contributing to the “Olive di Gaeta” processing style are very limited [24]. In terms of final pH, our data are consistent with previous evidence indicating that a 30-day fermentation followed by salt addition led to a pH of 4.2 [25]. Our work added that limiting the duration of the water-only fermentation stage produces slightly lower acidity. In comparison with the globally popular Spanish-style fermentation, the ‘Itrana’ method uses a reduced concentration of salt. Specifically, Spanish-style table olives are first treated with 1.8–2.5% (*w*/*v*) NaOH, washed, and then stored in a 10–13% NaCl solution [26]. Another difference is that Spanish-style fermentation is also shorter, with the second phase carried out at a minimum of 10–15 days before commercialization. The acidity equilibrium with the ‘Itrana’ method occurred in months, which is different from the Spanish method. While our results imply that lowering the salt concentration may not strongly affect the second phase of the ‘Itrana’ method, the W30-MS treatment also indirectly suggests that further lowering the NaCl percentage may be less effective in stabilizing the pH of the brine, potentially leading to an increased risk of microbial spoilage [27].

### 2.2. Evolution of Bacterial and Yeast Communities during Fermentation

**Figure 2:**
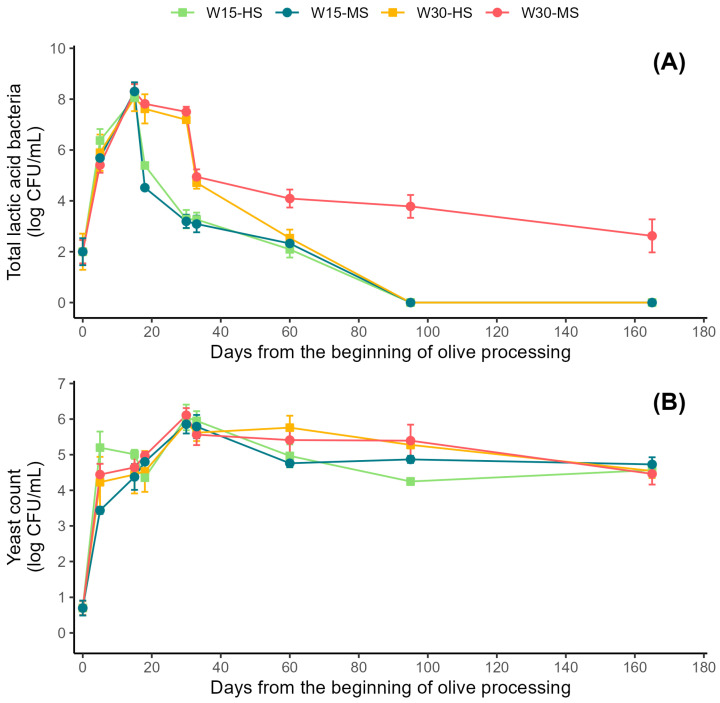
Microbiological changes in the brine. Evolution of total lactic acid bacteria count (**A**) and yeast count (**B**) during the four processing methods. W15-HS: 15 days in water and then salting with 8% NaCl brine (pea); W15-MS: 15 days in water and then salting with 6% NaCl brine (teal); W30-HS: 30 days in water and then salting with 8% NaCl brine (orange); W30-MS: 30 days in water and then salting with 6% NaCl brine (crimson).

Soon after the beginning of the storage period in water, both the bacterial and yeast counts rose (Figure 2). LAB growth was strongly influenced by the salt concentration and the time of the addition of the saline solution. In particular, the bacterial count reached the maximum value soon after the saline treatment (i.e., at 15 days or at 30 days), irrespective of the salt concentration that was added. Nonetheless, it is worth noting the distinctive dynamics of the W30-MS treatment. Only in this condition was the bacterial population significantly higher at the end of the fermentation process, while for the other three treatments, the bacteria count reached very low values starting 90 days from the beginning of the experiments.

Differences in yeast counts were much more limited. In particular, the yeast count was not significantly affected by the time of the use of the saline solution, nor by the salt concentration. The yeast population reached its maximum plateau value at 30 days, without significant differences at the end of olive processing.

The growth of microorganisms occurred rapidly when the olives were immersed in water in open tanks, and this strongly contributed to a rapid pH reduction [22]. A larger population of lactobacilli was recorded soon after the first two weeks of fermentation, as well as when salt was added at 30 days. This is a slightly later time compared to the Spanish method (7–10 days) [1]. A strong increase in LAB and yeasts is also typical of the Spanish and Greek trade preparation of green and fully ripe black olives, respectively [9,28]. Specifically, maximum growth occurs for the latter in less than one week because olives have been in brine since the beginning of fermentation [9]. Although yeast growth rose alongside LAB in the first natural, unsalted fermentation, in our samples, it presented a distinct growth profile at the later stages (i.e., after 90 days), regardless of the treatment [9]. This represents a notable feature of the ‘Itrana’ method, characterized by a predominance of yeasts over LAB only in the second half of the fermentation process. In the case of the natural fermentation of Greek-style black olives, the large contribution of yeasts is credited to a high salt concentration (10–14% *w*/*v*) [9,29]. In another study on Greek-style black olives, lowering the salt concentration to below 8% provided a similar effect on LAB, regardless of the temperature tested [30]. This illustrates the predominant importance of the salt concentration in controlling LAB growth. It is known that the response of microorganisms to NaCl, and other stressful conditions, is not linear. Moreover, there are species-specific differences [31], and therefore, a more detailed characterization of the LAB and yeast populations is necessary to unravel the reasons for the difference between W30-HS and W30-MS. In addition, nutrient availability may be another factor involved, since it is expected to be different at the two salting times (15 and 30 days of natural fermentation) [32]. Finally, the effects on LAB growth should also consider the microbial response to the inhibitory compounds present in the brines (e.g., polyphenols) at 15 and 30 days [33], as well as the interaction between polyphenols and salt concentration [34].

Phenolic compounds of ‘Itrana’ table olives underwent strong changes during fermentation. This parameter was influenced by the salt concentration and fermentation time (Table 1). After five days in water since the beginning of the preparation (D5), the most abundant phenolic compounds were two hydroxytyrosol derivatives (indicated in Table 1 as OHTy-der-1 and OHTy-der-2), luteolin-7-glucoside, verbascoside, and hydroxytyrosol glucoside (OHTy-glucoside). After 15 days in water and right before the salt application (D15), the levels of hydroxytyrosol derivatives and verbascoside in the olives significantly decreased compared to D5, while the concentrations of tyrosol-glucoside (Ty-glucoside), isoverbascoside, rutin, and luteolin slightly increased. At the end of processing (165 days from the beginning), W30-HS olives had significantly higher concentrations of OHTy-glucoside, Ty-glucoside, and luteolin compared to the beginning of processing (D5). Conversely, in the same period, other compounds decreased significantly, particularly vanillic acid, luteolin-7-glucoside, OHTy-derivatives, and OHTy-EA.

The compounds in our samples are consistent with the literature, including the degradation of biophenols and debittering during the fermentation process of table olives [35], although a large variation in commercial products is often reported [36]. For instance, a decrease in oleuropeins is associated with an increase in non-bitter hydroxytyrosols [37]. The latter rapidly diffuses into the brine, as also indicated by the difference between D5 and D15 [35]. It is well known that table olives are a source of multiple biophenols, and our data confirmed that their composition and concentration greatly vary not only according to the processing style [35] but also to the alteration of technical factors.

Interestingly, the reduction in the salt concentration was not associated with a lower oleuropein content in olives at commercial maturity. Its amount was also not influenced by the length of the first-stage water fermentation. The reduction in the salt concentration from 8 to 6% only caused a small decrease in iso-verbascoside, verbascoside, rutin, and luteolin-7-glucoside levels. In the case of olives fermented in water for 15 days before salt addition (W15-HS and W15-MS), OHTy-glucoside also decreased significantly in 8%-salted olives compared to those salted with 6% brine. In olives salted with 8% brine (W30-HS and W15-HS), small changes were observed in the phenolic content, particularly an increase in OHTy-glucoside and a decrease in the luteolin concentration when the duration of fermentation in water was increased from 15 to 30 days. The same trend was observed for 6%-salted olives for OHTy-glucoside. In this case, the level of luteolin was unaffected, and verbascoside seemed to increase as a longer fermentation time was applied. Caffeic acid was only detected in fermented samples, and its level did not significantly change in the four treatments.

The oleuropein content varies widely according to various environmental and genetic factors, as well as trade preparations, from a minimum of 1 mg/kg in black olives after storage to 504 mg/kg in fresh green olives [35]. Our findings showed a strong decrease after the first fermentation, indicating the predominant role of natural fermentation in the debittering of ‘Itrana’ olives.

To verify the type and concentration of phenolic compounds released in the liquid medium, we analyzed the brines of various treatments (Table 2). This can also reveal the release of phenolic compounds from the drupes to the brine. As expected, a strong increase in some phenolic compounds was observed in the brine during the first two weeks of fermentation (between D5 and D15), particularly OHTy-glucoside. Other compounds initially not detected in the olive brine (i.e., on D5) were successfully quantified on day 15. Independently of the salt concentration, OHTy-glucoside tended to decrease when the fermentation time was increased from 15 to 30 days. The levels of other phenolic compounds were generally not affected by the fermentation time or salt concentration, except for verbascoside and luteolin-7-glucoside, which were more abundant when the brine had 8% NaCl (W30-MS and W15-MS) compared to the other two processing methods.

The data indicated that the ‘Itrana’ method is not able to completely remove oleuropein from drupes, which explains the slightly bitter sensation typical of ‘Oliva di Gaeta’ (see also Section 2.4 describing the sensorial profile). For the Spanish trade preparation, the predominant role in the very high oleuropein removal is ascribed to the lye treatment and subsequent washes [24]. On the other hand, the ‘Itrana’ method, at the cost of a longer curing time, is characterized by a relatively mild alteration in the profile of the polyphenols in drupes. This is also consistent with another traditional Istrian two-stage fermentation process (water followed by saline solution), which better preserved the polyphenol content in the drupes compared to a Spanish-style preparation that included an alkaline treatment [38].

### 2.3. Effects on Volatile Organic Compounds (VOCs)

A total of 60 VOCs were identified by SPME-GC/MS analysis in fermented samples of ‘Itrana’ table olives, and semi-quantitative data are reported in Table 3.

In the ‘Itrana’ table olive headspace, in quantitative terms, high contents of ethanol, acetic acid, ethyl acetate, and 2-butanol were found in all samples, as expected [39]. The presence of ethanol, acetic acid, and other alcohols and esters indicated that our samples underwent alcoholic and heterolactic fermentations [39]. A strong difference was observed between the earliest-stage (D5) olives and the final products. For instance, in qualitative terms, we detected 37 volatile compounds in the headspace of D5 olives and from 53 to 56 compounds in the four treatments at the end of processing. Quantitatively, 29 volatile compounds of the D5 olives also shared with the trade preparation were present in significantly lower amounts than in at least one treatment. Decenal was the only volatile present in a higher quantity on D5 than trade preparations, and hexanal and nonenale were the only ones specific to this condition. It is likely that these lipid-derived volatiles are a sign of the start of the degradation process of the drupe [40] and therefore become irrelevant at the later stages of fermentation. The differences among olives at the end of the treatment were limited in qualitative terms. Specifically, most of the compounds (*n* = 52) were detected in all four samples. Treatment-specific volatiles (i.e., only in one trade preparation) were limonene and 2-undecenal, detected in relatively low amounts only in W30-MS olives. Overall, samples treated with the highest saline concentration (W30) had the highest relative quantities of most compounds. Conversely, 26 out of the 59 volatile compounds detected in W30-MS were present in statistically lower amounts compared to at least one of the other three preparations. It has been proposed that VOCs are detected in higher amounts in olives treated with higher NaCl concentrations due to a “salting out” effect, which promotes their transfer from the aqueous phase to the hydrophobic phase of the olives [41]. None of the detected compounds was present in a statistically higher amount exclusively in one table preparation. Moreover, the data indicate that the richer volatile profile of olives is obtained with high-salt treatments, while the combination of low salt and early saline treatment provides a more distinct profile. Overall, the qualitative differences among trade preparations were limited, confirming that most of the volatilome of the table olives is related to the fermentation process.

In comparison with other reports, 1-propanol, propyl-acetate, ethyl propanoate, and propionic acid were detected in higher levels in Spanish-style olives [11,42]. cis-3-Hexen-1-ol and 2-butanone were detected mainly in Castelvetrano-style olives [41], while 1-hexanol and isopentanol were found in higher concentrations in Spanish- and Greek-style olives [41,43]. Although generalizations have limitations for the food volatilome, treatment with sodium hydroxide, typical of Spanish-style processing, is often associated with p-creosol, phenylethyl alcohol, acetic acid, ethanol, benzyl alcohol, ethyl acetate, and (Z)-3-hexen-1-ol, with the amount of (E)-2-decenal significantly affected by the genotype [44]. Moreover, the Spanish-trade preparation is characterized by a higher content of acids (e.g., acetic acid and propionic acid), which was linked to predominantly acetic microflora [11].

SPME-GC/MS analysis was also carried out on the brines of the same treatments. In these samples, a total of 70 compounds were identified (Table 4). We detected 57 volatile compounds in the brine of D5 and from 50 to 56 in the four brines. However, the brine on D5 had 11 unique volatile compounds (i.e., not present in any other brines at commercial maturity). Regarding the differences among the brines of the four preparations, we observed both quantitative and qualitative variations.

Specifically, many of the compounds (*n* = 50) were shared (i.e., detected in all treatments), and only camphene was detected in one olive’s brine sample (W15-MS) in a limited amount (approx. 10%) compared to the D5 brine. The W30-HS brine had relatively higher amounts of compounds compared to the other treatments, while both the W15-HS and W15-MS brines most often had shared compounds in statistically lower amounts compared to the other conditions, suggesting that the time of the addition of the salt has a substantial impact on the volatiles in ‘Itrana’ style brines.

Overall, our data indicated that the influence of the experimental factors under investigation (time of salt application and salt concentration) on the volatile profile of mature olives was not largely condition-specific, with relatively limited statistical differences among trade preparations. We did not perform time-course monitoring of the samples, and therefore, we cannot exclude that those differences could be larger at intermediate stages of fermentation. Nonetheless, the limited effect of the experimental factors is consistent with the observed variations in the chemical and microbiological profiles. Finally, it is also necessary to mention that different salt concentrations in the brines can also represent a technical reason for analytical differences (e.g., a salting-out effect), as previously discussed for olives [45,46] and other food products [47]. The salting-out effect can contribute to the higher concentration of some volatile compounds in brines with higher salt concentrations.

### 2.4. Sensory Analysis

**Figure 3:**
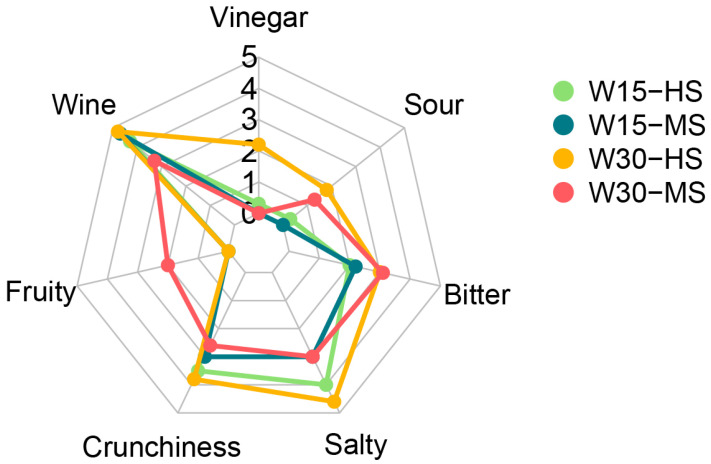
Sensory profile of ‘Itrana’ table olives at the end of four 165-day-long processing methods. For each treatment, the graph reports the median intensity for the different sensory attributes using a non-structured scale, starting from 0 (absence of the sensation) and ending at 10 (extremely intense). W15-HS:15 days in water and then salting with 8% NaCl brine (pea); W15-MS: 15 days in water and then salting with 6% NaCl brine (teal); W30-HS: 30 days in water and then salting with 8% NaCl brine (orange); W30-MS: 30 days in water and then salting with 6% NaCl brine (crimson).

To obtain an objective description of the sensory properties of the four trade preparations, we built a sensory profile according to the indications of the International Olive Council (IOC/OT/MO 1) without assessing visual features (brine color, skin color, and olive stone color) (Figure 3).

As expected, the higher salt concentration (8%) was associated with a higher “salty” score. The time of the addition of the salt had a very limited influence on the two 8% salt treatments, while differences were not noted by the panelists for the lower saline concentration (6%). Similarly, differences in crunchiness due to salt were limited, with slightly lower values with the 6% salt concentration compared to the 8% treatments. This attribute was unaffected by the time of the salt application. Differences due to the time of the application of salt were noted for the “sour” and “bitter” sensations, which were stronger for the W30 treatments, irrespective of the salt concentration. This can be explained by the fact that salt application strongly controls (microbiological) acidification and debittering [30]. For instance, the increasing role of (salt-tolerant) yeasts that follow the addition of salt may impart less of a “sour” perception to the olive [48]. It should be added that the addition of the highest salt dose at the later time (W30-HS) was coupled with a stronger “vinegar” perception compared to the other three treatments. Moreover, it is interesting that olives obtained with the W30-MS condition had a higher score for the “fruity” attribute than all other preparations. W30-MS had a more distinct microbiological profile, characterized by a slower decline in LAB (Figure 2A). Although the relation between LAB and flavor development is complex, it is possible to speculate that the more “fruity” description may derive from ethyl esters, probably derived from the reaction of ethanol and free fatty acids and further conversions [49].

For ‘Itrana’ olives, it has been shown that a fermentation of 30 days in water followed by brine resulted in higher scores for “bitterness” and significantly lower scores for “acid” [22]. Our results are in partial agreement with previous research because the bitterness score was higher at both salt concentrations, while samples treated at 30 days had higher scores for “sour” [22]. This difference might be due to the development of abnormal fermentation in the longer fermentation time reported by Lanza et al. [22].

## 3. Materials and Methods

### 3.1. Olive Sampling and Experimental Design

Olives of the cultivar ‘Itrana’ were harvested in a commercial olive grove located near the town of Itri (Latina, Italy) in April. Olives with a diameter larger than 14 mm were selected and immediately transported to the facilities of the Cooperativa Unione Agricoltori Itrani (Itri, Latina, Italy), where the processing was carried out. Within a few hours after harvest, 12 polyethylene containers for olive fermentation (190 L volume) were each filled with 125 kg of selected olives. Fermentation consisted of two consecutive stages. During the first stage, olives were immersed in water and were stored in open containers. For the second stage (salting), water was replaced by brine, and the containers were closed (the tank was completely filled with saline solution to reduce the available headspace and ensure anaerobic conditions).

The experiment compared four treatments derived from the combination of two durations of the first fermentation stage (15 or 30 days in water; W15 and W30, respectively) and two NaCl concentrations in the brine used for the second fermentation stage: 6% NaCl added to obtain a final concentration of about 4% NaCl at commercial maturity (MS), and 8% NaCl added to reach a final NaCl concentration of 6% NaCl at commercial maturity (HS). The treatments were therefore the following: (i) 30 days in water and then salting with 8% NaCl brine (W30-HS); (ii) 15 days in water and then salting with 8% NaCl brine (W15-HS); (iii) 30 days in water and then salting with 6% NaCl brine (W30-MS); (iv) 15 days in water and then salting with 6% NaCl brine (W15-MS). Three different fermentation containers (tanks) were used per treatment. Independently of the treatment, the whole fermentation process (first stage in water plus second stage in brine) lasted a total of 165 days.

### 3.2. Physical–Chemical Characteristics of the Drupes Used in the Experiment

To characterize the physical-chemical characteristics of the olives selected for the experiment, 30 olives with a minimum diameter of 14 mm were randomly sampled before putting them in the plastic containers. The following parameters were measured: fruit diameter and length, fruit fresh weight, flesh and pit fresh weights, and moisture content. The latter parameter was evaluated gravimetrically after drying the olives in a thermostatic incubator at 105 °C until reaching a constant weight.

### 3.3. Quantification of Lactic Acid Bacteria and Yeast Populations during Fermentation

Each sample (10 g of olives from the four preparations and 25 mL of the corresponding brine, collected at 0, 5, 15, 18, 30, 33, 60, 95, and 165 days) was added to 315 mL of a 0.9% NaCl solution and homogenized with a sterile circulator blender (Stomacher 400; Seward Medical, London, UK) for two minutes. Samples were serially diluted and plated in triplicate, as previously reported [48,50]. Growth data from plate counts are presented as log10 values per mL.

### 3.4. Measurements of pH and Salt Concentration during Fermentation

The pH of the brine was measured on 14 dates during fermentation by using a pH-meter (S20 SevenEasy pH; Mettler Toledo, Milan, Italy), whereas the salt concentration in the brine was measured on eight dates by titrating with AgNO_3_ 0.10 molL^−1^ AgNO_3_ using a 0.25 molL^−1^ K_2_CrO_4_ solution as an indicator (Mohr’s method).

### 3.5. Analysis of Phenolic Compounds in Olives and Brine

Phenolic compounds in the olives and brine were measured on two dates during the first fermentation stage (5 and 15 days from the beginning of the experiment, when the olives of all treatments were in water) and at the end of the fermentation process (165 days from the beginning of the experiment).

Phenolic compounds were extracted according to a previously described method [8] with some modifications. Briefly, olive pulp (10 g) from at least 10 olive drupes was collected and crushed by using an Ultraturrax T25 blender (Ika, Staufen, Germany) after the addition of a methanol–water solution (80:20). Just before the procedure, 400 ppm sodium metabisulfite was added to the solution to prevent possible oxidation. The homogenate was centrifuged for 5 min at 4000 rpm (ALC centrifuge mod. PK120). The hydro-alcoholic extract was filtered and collected, while the olive pulp residue was washed another three times by using a 90 mL total volume of the methanol–water solution. All extracts were collected and evaporated under vacuum in a rotary evaporator (Heidolph mod. VV2000 (Schwabach, Germany)) at 35 °C until the methanol was evaporated. A total of 20 mL of distilled water was added, and the solution was washed 3 times by using 15 mL of hexane for a final volume of 45 mL. One milliliter of syringic acid (200 ppm) was added to this solution as an internal standard. The extraction of phenolic compounds was carried out by using ethyl acetate 5 times with a total volume of 100 mL. The solvent was evaporated in a rotary evaporator at 25 °C, and the residue was dissolved in 2 mL of methanol. An aliquot (25 μL) was used for HPLC analysis. A total of 5 mL of olive brine was taken, and 0.5 mL of syringic acid was added as an internal standard (200 ppm). The solution was evaporated at 35 °C and dissolved by using 2 mL of methanol.

The analysis of phenolics was carried out as previously reported [51]. The HPLC eluent phases were as follows: (A) water–TFA (97:3) and (B) methanol–acetonitrile (20:80). Gradient elution started with 5% solution B to reach 60% B at 35 min. A Shimadzu LD-10ADVP HPLC equipped with a UV-Vis DAD Shimadzu SPD-M10AVP (Shimadzu, Milan, Italy) was used, and a Spherisorb S5 ODS3 250 × 4.6 mm i.d. reversed-phase column was used. The column flow was 1 mL min^−1^, and the injected volume was 20 μL. Detection wavelengths were 279 and 239 nm. Syringic acid was used as an internal standard. For phenolic compound identification, a parallel HPLC analysis was carried out with a similar instrument equipped with an MS detector (Shimadzu MS-2010EV, Milan, Italy). HPLC conditions were the same as described above, and the MS conditions were as follows: CDL temperature: 300 °C; gas flow: 1.5 KW; interface voltage: 0.3 KV; RF: 120 V; acquisition interval: 60–900 *m*/*z*.

### 3.6. Volatile Organic Compounds in Olives and Brine

The volatile compounds in the olives and brines were measured at the end of the fermentation process (165 days from the beginning of the experiment). This analysis was carried out by using the SPME-GC/MS technique, as described in [44], with some slight modifications. Olive pulp (3 g) was taken from a minimum of five olives, and it was crushed and homogenized by using an Ultra Turrax T25 blender (Kilka-Werke, Staufen, Germany) for about 1 min after the addition of 5 mL of NaCl solution (300 g L^−1^) distilled water. The homogenate (3 mL) was added to a 15 mL vial, and 3 μL of isobutyl acetate (2000 ppb, Sigma-Aldrich, Steinheim, Germany) was added as an internal standard. The vial was heated at 30 °C for 10 min for the equilibration phase under stirring. A fiber was exposed to the sample for 30 min at the same temperature. A Supelco (Bellefonte, PA, USA) DVB-CAR-PDMS 50/30 μm fiber was used. The fiber was then injected into a Shimadzu QP5050 (Milan, Italy) GC-MS chromatographer for 10 min at 230 °C. The column was a 60 m × 0.32 mm, 0.5 μm SupelcoWAX 10 (Supelco, Bellefonte, MA, USA). Conditions used for GC were as follows: Helium was used as the carrier gas, the flow rate was 1.4 mL min^−1^, the initial pressure was 52 KPa, and the initial column temperature was 40 °C. An isotherm was maintained at 40 °C for 4 min, and then a rate of 3.5 °C min^−1^ was applied until 240 °C, which was held for 3 min. MS conditions were as follows: ionic source using electron impact of 70 eV, interface temperature of 250 °C, ionic source temperature of 200 °C, mass range of 30–250 amu, and scan velocity of 0.4 scans s^−1^. Compound identification was performed by comparing mass spectra and retention times with those of pure compounds used as a reference, when available. In other cases, a tentative identification was made by using mass spectra libraries (NIST 27, NIST 147, and SZTERP) found in the acquisition software, with a similarity higher than 85%. Olive brine was analyzed as described for olive drupes, but the sample volume was 3 mL, and 6 μL of 3-pentenol (1000 ppb) was added as an internal standard. The analysis of volatile compounds was carried out by using the total ion current (TIC) modality.

### 3.7. Sensory Analysis of Olives

The sensorial attributes of the olives were evaluated at the end of the fermentation process (165 days from the beginning of the experiment). A quantitative descriptive method was used for ‘Itrana’ table olive sensory analysis, according to the International Olive Council (IOC) method for table olives (https://www.internationaloliveoil.org/olive-world/table-olives/ accessed on the 1 October 2022). A ten-member panel was employed, and panelists were selected among students and scholars at the University of Naples Federico II (Italy) and from ‘Camera di Commercio di Napoli’ (Italy). Panelists were trained in preliminary sessions by using different samples of table olives to develop a common vocabulary for the description of the sensory attributes of the experimental samples. Each attribute was described and explained to avoid doubts about its meaning. Based on the frequency of citation (>60%), seven descriptors were added to the profile sheet: vinegar, winey, fruity, crunchiness, salty, bitter, and sour. Random samples were evaluated by using a non-structured scale, starting from 0 (absence of the sensation) and ending at 10 (extremely intense). Each sampling glass contained about 7–10 olives, and the analysis was carried out in triplicate. Four samples were analyzed for each session, and 50 mL plastic glasses were used.

### 3.8. Statistical Analysis

All analyses were performed in triplicate. Significant differences between treatments were determined for each measured trait with one-way ANOVA (*p* < 0.05) using Tukey’s test to discriminate among the means. Data analyses were carried out using XLStat, an add-in software package for Microsoft Excel (Addinsoft Corp., Paris, France).

## 4. Conclusions

This work investigated the impact of the key processing factors on a wide range of properties (i.e., physico–chemical, microbiological, and sensory scores) of the most popular Italian table olive. To our knowledge, this is the first time that such a comprehensive analysis was performed on the ‘Itrana’ trade preparation olives and their brines.

The fermentation of “Oliva di Gaeta”, based on indigenous microflora, is an element of the *terroir* for this local olive variety. Our work illustrated the effects of two technical factors that define the two-stage trade preparation of ‘Itrana’ olives on their chemical, microbiological, biochemical, and sensorial features. Considering the magnitude of the observed effects, the knowledge generated will enable the direction of the selection of conditions that may be exploited to improve the nutritional (e.g., amount of polyphenols), sustainability (e.g., use of salt), and sensorial qualities (e.g., bitterness and fruitiness) of ‘Itrana’ olives, with the 6% NaCl treatment after 30 days of water fermentation being the most different condition in terms of microbiological dynamics, polyphenols, and sensory evaluation. This information will be useful to the food industry for standardizing and optimizing their processing and for guaranteeing a safe and stable production.

## Figures and Tables

**Table 1:** Phenolic compounds analyzed by HPLC-DAD in ‘Itrana’ table olives after 5 and 15 days of fermentation in water (D5 and D15, respectively) and at the end of four 165-day-long processing methods: 30 days in water and then salting with 8% NaCl brine (W30-HS); 15 days in water and then salting with 8% NaCl brine (W15-HS); 30 days in water and then salting with 6% NaCl brine (W30-MS); 15 days in water and then salting with 6% NaCl brine (W15-MS). Within each column, different letters indicate significant differences between treatments according to Tukey’s test (*p* ≤ 0.05). Values are expressed as mg internal standard per kg of olive pulp (*n* = 3).

Treatment	OHTy-Glucoside	Ty-Glucoside	Caffeic Acid	Vanillic Acid	Iso-Verbascoside	Verbascoside	Rutin	Luteolin-7-Glucoside	OHTy-der-1	OHTy-der-2	Oleuropein	Luteolin	OHTy-EA
D5	102.8 c	9.2 c	nd	25.8 a	31.3 b	142.2 a	71.2 b	125.1 a	93.0 a	152.8 a	83.1 a	66.0 c	52.1 a
D15	102.3 c	15.3 b	nd	17.4 b	52.4 a	102.7 b	86.4 a	79.0 b	85.6 a	86.1 b	75.5 b	95.5 b	40.3 a
W30-HS	216.3 a	16.5 b	18.6 b	17.4 b	52.5 a	93.2 bc	81.1 a	55.4 c	33.3 b	19.4 c	30.2 c	96.2 b	16.4 b
W15-HS	195.9 b	20.6 ab	20.8 a	16.4 b	44.9 a	85.1 bc	84.7 a	56.3 c	27.4 b	18.0 c	32.0 c	109.4 a	14.2 b
W30-MS	198.6 b	21.3 a	18.2 b	16.8 b	42.6 a	75.3 c	65.7 b	48.1 d	31.0 b	21.0 c	29.1 c	101.5 a	13.0 b
W15-MS	167.1 bc	21.5 a	20.9 a	17.1 b	36.6 ab	58.8 d	77.8 ab	48.1 d	32.0 b	19.0 c	37.4 c	100.4 a	13.5 b

OHTy: hydroxytyrosol; Ty: tyrosol; der.: derivative; nd: not detected.

**Table 2:** Phenolic compounds analyzed by HPLC-DAD in the brine used to process ‘Itrana’ table olives after 5 and 15 days of fermentation in water (D5 and D15, respectively) and at the end of four 165-day-long processing methods: 30 days in water and then salting with 8% NaCl brine (W30-HS); 15 days in water and then salting with 8% NaCl brine (W15-HS); 30 days in water and then salting with 6% NaCl brine (W30-MS); 15 days in water and then salting with 6% NaCl brine (W15-MS). Within each column, different letters indicate significant differences between treatments according to Tukey’s test (*p* ≤ 0.05). Values are expressed as mg internal standard per L of brine (*n* = 3).

Treatment	OHTy-Glucoside	Ty-Glucoside	Caffeic Acid	Vanillic Acid	Iso-Verbascoside	Verbascoside	Rutin	Luteolin-7-Glucoside	OHTy-der-1	OHTy-der-2	Oleuropein	Luteolin	OHTy-EA
D5	7.7 d	3.4 d	nd	nd	nd	nd	nd	nd	3.9 d	2.5 c	nd	8.2 c	nd
D15	206.5 c	33.7 c	nd	22.5 c	17.3 c	24.5 d	16.4 b	45.0 c	30.9 c	44.4 a	30.0 a	17.0 b	nd
W30-HS	645.3 b	72.1 b	40.7 b	43.2 b	51.5 b	98.5 c	72.0 a	74.3 b	49.1 b	29.7 b	24.2 a	21.3 b	nd
W15-HS	725.2 ab	73.5 b	50.0 ab	48.8 b	52.5 b	104.1 b	81.4 a	100.2 ab	55.6 b	30.7 b	22.3 a	26.2 b	nd
W30-MS	728.7 ab	86.0 a	52.1 ab	54.5 a	69.8 a	122.9 a	55.3 ab	135.8 a	63.9 b	31.5 b	27.9 a	30.0 ab	nd
W15-MS	807.3 a	87.0 a	67.9 a	55.9 a	61.9 a	128.1 a	78.0 a	132.1 a	76.0 a	33.1 b	24.6 a	34.4 a	nd

OHTy: hydroxytyrosol; Ty: tyrosol; der.: derivative; nd: not detected.

**Table 3:** Volatile compounds in ‘Itrana’ table olives processed by using different salt concentrations and fermentation times. Data were taken after 165 days from the beginning of the process, except for D5 (olives five days following the beginning of the treatment). Samples are as follows: 30 days in water and then salting with 8% NaCl brine (W30-HS); 15 days in water and then salting with 8% NaCl brine (W15-HS); 30 days in water and then salting with 6% NaCl brine (W30-MS); 15 days in water and then salting with 6% NaCl brine (W15-MS). Within each row, different letters indicate significant differences between treatments according to Tukey’s test (*p* ≤ 0.05). Values are expressed as µg internal standard per kg of olive pulp (*n* = 3).

Volatile Compound	I.M. *	D5	W30-HS	W15-HS	W30-MS	W15-MS
Acetaldehyde	RT	nd	83 a	116 a	nd	nd
Dimethyl sulfide	TI	30 b	104 a	91 a	73 ab	54 ab
Octane	RT	41 b	437 b	1472 a	810 ab	882 ab
2-Methylpropanal	RT	7 b	25 a	15 ab	12 b	12 b
Methyl acetate	RT	4 b	71 a	64 a	51 ab	42 ab
Octene	RT	7 b	88 a	110 a	77 ab	94 a
Ethyl acetate	RT	28 b	2039 a	1751 a	1481 a	1256 ab
Methanol	RT	28 b	417 a	344 a	366 a	267 ab
2-Methylbutanal	RT	20 b	121 a	82 ab	68 ab	68 ab
3-Methylbutanal	RT	17 b	139 a	84 ab	75 ab	74 ab
Ethanol	RT	290 b	12,648 a	13,618 a	11,031 a	9467 ab
Ethyl isobutyrate	RT	nd	35 a	30 ab	36 a	24 b
2-Pentanone	RT	5 b	10 ab	5 b	4 b	13 a
Ethyl butanoate	RT	nd	39 a	35 ab	19 b	28 ab
1-Propanol	RT	nd	18 a	18 a	9 b	18 a
Toluene	RT	6 b	8 a	7 ab	7 ab	8 a
Ethyl 2-methylbutanoate	RT	9 b	108 a	89 a	84 a	76 ab
Ethyl 3-methylbutanoate	RT	25 b	260 a	245 a	205 a	214 a
Hexanal	RT	119 ab	158 a	97 ab	123 a	51 b
Butyl 2-methylpropanoate	RT	nd	nd	nd	34 a	27 a
2-Methylpropanol	RT	nd	72 a	58 ab	56 ab	48 b
3-Methylbutyl acetate	RT	nd	151 ab	158 a	115 b	151 ab
1-Butanol	RT	nd	8 a	7 ab	6 ab	5 b
2-Heptanone	RT	nd	2 a	6 a	nd	3 a
Heptanal	RT	11 b	9 b	23 a	17 ab	13 ab
Limonene	RT	1 a	nd	nd	nd	10 a
3-Methyl-1-butanol	RT	15 b	1254 a	1060 a	1001 a	1092 a
(E)-2-Hexenal	RT	71	nd	nd	nd	nd
Ethyl hexanoate	RT	nd	149 ab	166 a	138 ab	77 b
(E/Z)-Ocimene	TI	45 b	105 ab	109 ab	134 a	60 b
Styrene	RT	nd	2306 b	3255 a	2608 ab	2677 ab
n-Hexyl acetate	RT	nd	14 b	29 a	25 ab	24 ab
Octanal	RT	22 a	7 b	8 ab	nd	6 b
4,8-Dimethylnona-1,3,7-triene	TI	3 b	9 ab	12 a	12 a	8 ab
(Z)-3-Hexenylacetate	RT	49 a	38 ab	39 ab	38 ab	27 b
Ethyl heptanoate	RT	nd	3 ab	8 a	7 ab	2 b
6-Methylhept-5-en-2-one	RT	5 b	26 a	30 a	23 a	21 ab
Hexanol	RT	60 b	291 a	269 a	304 a	180 ab
(Z)-3-Hexenol	RT	99 c	157 ab	156 abc	167 a	107 bc
Methyl octanoate	RT	nd	13 a	10 ab	12 a	4 b
Nonanal	RT	75 a	14 b	26 b	12 b	21 b
(E)-2-Hexenol	RT	17 a	3 b	3 b	3 b	6 ab
Ethyl octanoate	RT	nd	83 a	60 ab	91 a	29 b
Heptanol	RT	6 b	73 a	79 a	76 a	44 ab
2-Ethylhexan-1-ol	TI	6 b	25 a	27 a	26 a	23 a
6-Hepten-1-ol	RT	nd	14 ab	15 a	14 ab	9 b
Linalool	RT	nd	8 ab	10 a	9 ab	5 b
(E)-2-Nonenal	TI	5	nd	nd	nd	nd
Octanol	RT	12 b	34 ab	40 a	43 a	22 ab
9-Decenol	RT	nd	6 ab	8 a	4 ab	2 b
(E)-2-Decenal	RT	88 a	7 b	21 b	12 b	8 b
Nonanol	RT	nd	69 ab	81 a	87 a	47 b
Ethyl benzoate	RT	nd	9 ab	11 ab	12 a	7 b
Farnesene	RT	23 b	121 ab	142 a	188 a	86 ab
1-Decanol	RT	nd	9 ab	9 ab	12 a	6 b
2-Undecenal	RT	85 a	nd	nd	nd	4 a
2-Methoxyphenol	RT	nd	3 a	nd	2 a	2 a
Phenylmethanol	TI	2 b	20 ab	17 ab	30 a	13 ab
Ethyl 3-phenylpropanoate	RT	nd	9 a	9 a	9 a	5 b
2-Phenylethanol	RT	10 b	106 a	96 a	122 a	69 ab

* I.M., identification method: RT, comparison of mass spectra and GC retention times with pure reference compounds; TI, tentative identification by using mass spectra found in NIST libraries; nd: not detected.

**Table 4:** Volatile compounds in the brine of ‘Itrana’ table olives processed by using different salt concentrations and fermentation times. Data were taken after 165 days from the beginning of the process, except for D5 (olives five days following the beginning of the treatment). Samples are as follows: 30 days in water and then salting with 8% NaCl brine (W30-HS); 15 days in water and then salting with 8% NaCl brine (W15-HS); 30 days in water and then salting with 6% NaCl brine (W30-MS); 15 days in water and then salting with 6% NaCl brine (W15-MS). Within each row, different letters indicate significant differences between treatments according to Tukey’s test (*p* ≤ 0.05). Values are expressed as µg internal standard per L of brine (*n* = 3).

Volatile Compound	I.M. *	D5	W30-HS	W15-HS	W30-MS	W15-MS
Acetaldehyde	RT	nd	1198 a	989 b	1164 ab	1063 ab
Dimethyl sulfide	TI	44 b	281 a	209 ab	247 a	195 ab
Octane	RT	26 a	nd	nd	26 a	36 a
2-Methylpropanal	RT	nd	41 a	nd	40 a	46 a
Methyl acetate	RT	441 b	982 a	687 ab	905 a	639 ab
2,4-Dimethyleptene	TI	65 b	323 ab	189 ab	430 a	416 a
Ethyl Acetate	RT	10,647 b	18,142 a	14,160 ab	16,970 a	13,787 ab
Methanol	RT	1762 b	4841 a	4012 a	4018 a	3806 ab
2-Methylbutanal	RT	211 b	459 a	418 ab	461 a	554 a
3-Methylbutanal	RT	210 b	435 ab	373 ab	463 a	568 a
Ethanol	RT	51,713 b	126,636 a	113,363 a	105,356 ab	117,646 a
Ethyl propanoate	RT	nd	13 a	nd	299 a	nd
Ethyl isobutyrate	RT	74 ab	42 b	83 a	96 a	95 a
2-Pentanone	RT	63 c	122 ab	80 bc	99 abc	144 a
2-Methylpropyl acetate	RT	nd	61 a	48 b	60 ab	63 a
Ethyl butanoate	RT	92 ab	104 a	75 b	94 ab	106 a
1-Propanol	RT	nd	193 ab	213 ab	161 b	253 a
Ethyl 2-methylbutanoate	RT	144 ab	154 a	104 b	151 a	117 ab
Camphene	RT	243 a	nd	nd	nd	30 a
Ethyl 3-methylbutanoate	RT	275 b	557 a	323 b	339 b	295 b
Hexanal	RT	1072 a	191 b	362 ab	21 b	217 b
2-Methylpropanol	RT	79 b	1071 a	1008 a	981 a	1007 a
β-Pinene	RT	463	nd	nd	nd	nd
3-Methylbutyl acetate	RT	172 b	696 a	432 ab	664 a	529 ab
3-Carene	TI	15	nd	nd	nd	nd
3-Heptanone	RT	77 b	113.5 a	85.1 ab	74 b	85 b
Heptanal	RT	520 a	79.9 b	61.8 b	28 b	62 b
Limonene	RT	17,015	nd	nd	nd	nd
3-Methylbutanol	RT	917 b	13,807.2 a	11,604.3 a	13,051 a	14,641 a
Eucalyptol	TI	409	nd	nd	nd	nd
4-Methylheptan-2-one	RT	nd	251.3 ab	203.3 b	468 ab	557 a
Ethyl 3-methylbut-2-enoate	TI	15 b	58.3 a	21.4 b	28 ab	34 ab
Ethyl hexanoate	RT	530 a	237.1 ab	123 b	207 b	114 b
4,6-Dimethylheptan-2-one	TI	nd	23.2 a	nd	55 a	69 a
1-Pentanol	RT	46 b	98.8 a	70.7 ab	92 a	100 a
3-Methylbut-3-en-1-ol	TI	16 b	47.5 a	nd	44 a	27 ab
Styrene	RT	22 b	157.6 ab	166.9 ab	220 ab	335 a
Octanal	RT	895 a	141.1 b	115.4 b	76 b	103.2 b
1-Octen-3-one	RT	43	nd	nd	nd	nd
2-Methyl-2-octanol	RT	nd	64.2 ab	55.9 b	61 ab	66.6 a
(Z)-3-Hexenyl acetate	RT	68 ab	72.3 a	69.5 a	70 a	63.2 b
Ethyl heptanoate	RT	114	nd	nd	nd	nd
6-Methyl-5-hepten-2-one	RT	75 a	36 b	31.1 b	31 b	33.4 b
Ethyl 2-hydroxypropanoate	TI	nd	238.5 ab	125.4 b	333 a	183.6 ab
Hexanol	RT	754 b	1807.3 a	1422.1 ab	1792 a	1407.7 ab
(Z)-3-Hexenol	RT	713 b	1301.4 a	1029.3 ab	1272 a	1012.6 ab
Methyl octanoate	RT	26 a	61.3 a	nd	22 a	nd
Nonanal	RT	1487 a	557.8 b	467.7 b	279 b	381.6 b
(E)-2-Hexenol	RT	102 a	37 ab	nd	25 b	22.8 b
Ethyl octanoate	RT	268 a	215.4 ab	102.3 b	219 ab	75.2 b
(E)-2-Octenal	RT	67 a	nd	30.2 a	nd	30.3 a
1-Octen-3-ol	RT	150	nd	nd	nd	nd
Heptanol	RT	121 b	314.3 a	269.4 a	241 ab	247.8 ab
4-Octenoic acid, ethyl ester, (Z)-	TI	151 a	78.6 ab	49.2 b	70 ab	nd
Decanal	RT	612 a	105.8 b	81.7 b	76 b	113.3 b
6-Hepten-1-ol	TI	nd	90.3 ab	165.2 a	64 b	75 b
(E)-2-Hepten-1-ol	RT	99	nd	nd	nd	nd
2-Methyldecanal	TI	124	nd	nd	nd	nd
Ethyl nonanoate	RT	192	nd	nd	nd	nd
Benzaldehyde	RT	nd	119.4 a	64.5 b	95 ab	109.7 a
Linalool	RT	570	nd	nd	nd	nd
1-Octanol	RT	262 a	129.3 b	156.9 ab	91 b	104.6 b
(E)-2-Decenal	RT	125 a	70 ab	67 ab	nd	33.2 b
Nonanol	RT	217 a	202 ab	181 ab	143 b	144.1 b
4-Methyl benzaldehyde	TI	nd	50 ab	78 ab	41 b	82.9 a
2-Undecenal	RT	nd	63.1 a	53.4 ab	nd	42.9 b
Benzyl alcohol	RT	99 b	206.1 ab	294.2 a	192 ab	127.4 b
Ethyl 3-phenylpropanoate	TI	245 a	63.1 ab	nd	43 b	12.1 b
2-Phenylethanol	RT	201 b	796.3 a	731.9 a	537 ab	574.5 ab
1-Dodecanol	RT	48 b	53 b	146.2 a	119 ab	74.1 ab

* I.M., identification method: RT, comparison of mass spectra and GC retention times with pure reference compounds; TI, tentative identification by using mass spectra found in NIST libraries; nd: not detected.

## Data Availability

Data supporting the conclusions of this article not already presented in the tables and figures will be available from the corresponding author (R.S.) upon reasonable request.

## Sample Availability

Sample sharing is not applicable to this article as no new samples or compounds were created, purified, or isolated and used in biological activity assays in this study.
